# A Qualitative Longitudinal Study of Injuries and Medical Care, Assistance, and Losses Recounted by Oklahoma City Bombing Survivors after Nearly a Quarter Century

**DOI:** 10.1017/S1049023X22001133

**Published:** 2022-10

**Authors:** Carol S. North, Katy McDonald, Alina Surís

**Affiliations:** 1. Altshuler Center for Education and Research at Metrocare Services, Dallas, Texas USA; 2. Department of Psychiatry at the University of Texas Southwestern Medical Center, Dallas, Texas USA

**Keywords:** disaster experience, medical injuries, prospective longitudinal follow-up study, qualitative data, terrorism

## Abstract

**Introduction:**

Terrorist incidents occur with alarming frequency. Much is known about acute injuries and psychopathology arising from terrorism, as well as medical care and functional status assessed in early post-disaster periods. Survivors’ memories of these experiences may change over subsequent decades, and their perspectives may evolve. Little information is available on how survivors describe these experiences decades later.

**Study Objective:**

This longitudinal qualitative study of directly-exposed survivors of the 1995 Oklahoma City bombing was conducted nearly a quarter century after the disaster. It collected systematic, open-ended descriptions of survivors’ injuries and medical care, assistance received and given, and disaster-associated losses. It sought to illuminate whether survivors recall long-term consequences of disaster exposure so long after the event, providing important details with great clarity and associated emotion, or alternatively lose memory and sharpness of recollection for these aspects of their bombing experience.

**Methods:**

A sample of 182 bombing survivors was randomly recruited from a state registry of 1,092 bombing survivors and interviewed at approximately six months after the bombing (71% participation). The sample was re-interviewed an average of 23 years after the disaster (72% follow-up participation) using an open-ended interview with survivors describing in their own words their personal experience of the bombing and its effects on their lives. The interviews were audio recorded and professionally transcribed. Themes were identified in the text of the interviews, and passages were coded using qualitative software, achieving excellent inter-rater reliability for each theme. This article covers three of twelve total themes identified.

**Results:**

Nearly a quarter century after the bombing, this highly trauma-exposed Oklahoma City bombing survivor sample had memories that were still vivid, graphic, and evocative. They described injuries and medical care, assistance given and received, and losses with great detail and intensity. Despite the continuing strong emotions expressed by these survivors in relation to the bombing, the qualitative content suggested that lasting psychopathology was not a central concern.

**Conclusion:**

This is one of the longest prospective longitudinal, qualitative studies ever conducted with highly trauma-exposed survivors of a terrorist bombing. These findings are critical to disaster emergency response and effective management of the disaster response and early care for the survivors, as the effects of the disaster may shape the rest of their lives.

## Introduction

At 9:02am on April 19, 1995, a domestic terrorist detonated a homemade truck bomb in front of the Oklahoma City Murrah Federal Building (Oklahoma USA), blowing off the front-half of the building and severely damaging nearby structures, resulting in 168 fatalities. At the time, it was the most severe terrorist attack in United States history.

There is an extensive literature on acute injuries and psychopathology resulting from disaster trauma exposures, as well as medical care and functional status assessed in early post-disaster periods. Over the decades following disaster trauma exposure, memory of these disaster experiences may change, survivors’ personal perspectives on the bombing may progress, and descriptions of injuries, medical care received, and functioning may evolve over time. There exists very little information on the long-term impressions of terrorism survivors’ injuries, medical care, and functional status from a longitudinal perspective of more than a decade.

One long-term study of injuries, mental health (MH) problems, and functioning conducted 14 years after the September 11, 2001 (9/11) attacks on New York City’s World Trade Center towers (New York USA) collected qualitative data from semi-structured interviews of injured survivors recruited from a registry.^1^ Injuries were sustained by 44%, largely consisting of fractures and dislocations, burns, and head injuries. Injuries strongly predicted MH problems, especially posttraumatic stress. Many survivors received medical care, including care for critical or serious conditions. Long-term disability was especially associated with orthopedic injuries. Most survivors reported long-lasting changes in their lifestyles and many suffered negative economic consequences.

Numerous post-disaster studies focused on MH have conducted prospective examinations of survivors’ long-term MH outcomes using quantitative methods.^2–16^ A large proportion of this research designated as “long term” has followed survivors for only ≤four years or even five-to-nine years; however, follow-up durations of ≥ten years are of the greatest relevance to the current study. Quantitative studies of MH outcome trajectories from early post-disaster periods up to 10-27 years have found that psychopathology, emotional distress, and impaired functioning including employment difficulties may generally improve across time, but for some survivors, these difficulties may continue for long periods.^2–17^

Almost no longitudinal research using qualitative methods as long as a decade later has been conducted to determine how surviving a terrorist attack affects people’s lives long term with the richness and detail that qualitative data can provide. Open-ended interviews allow participants to recount their experience in their own words, unconstrained and undirected by the interviews, allowing collection of information beyond data from quantitative interviews limited to restricted categories. One prior long-term study of Oklahoma City bombing survivors examined a different sample from the same registry as used for the current study 18.5 years post-disaster; 81% of the sample was injured in the bombing. This study collected both quantitative and qualitative data.^6,18^ The quantitative findings reflected large numbers of disaster-related injuries in general, especially hearing loss, with brief mention of musculoskeletal injuries, pain, head and eye injuries, lacerations, and pulmonary injuries, but with detail limited to stating the types of injuries. Fewer than one-half of those survivors reported physical injuries and health problems, posttraumatic stress symptoms and other emotional difficulties, or psychosocial or interpersonal problems, with little detail and few illustrative quotations. This lack of detail may relate to the method of inquiry, in which survivors were asked to name the “three most important problems” and the “three current most important needs” they had experienced as a result of the bombing, constraining the qualitative data to only three responses.^18(p.3)^

Much more needs to be known about the medical and psychosocial consequences and perspectives on the disaster experience, such as assistance received and given and effects of personal losses in disaster over long periods of time afterwards. Terrorist incidents have been occurring with alarming frequency, and thus survivors of these events represent a substantial population that will carry this experience with them for the rest of their lives. Therefore, this longitudinal qualitative study of a sample of directly-exposed survivors of the Oklahoma City bombing was conducted nearly a quarter century after the disaster to generate long-term systematic information from open-ended descriptions of disaster survivors regarding their injuries, medical care, assistance received and given, and disaster-associated losses. This study sought to determine whether survivors will report consequences of disaster exposure as long as nearly a quarter century afterward, and if they still recall important details with great clarity and associated emotion, or alternatively have lost memory and sharpness of recollection for these aspects of their bombing experience.

## Methods

A sample of 182 bombing survivors was randomly recruited from a state registry of 1,092 bombing survivors and assessed approximately six months after the bombing, with a 71% participation rate. Details of the original study methods and characteristics of the sample and psychiatric disorders are provided in an earlier publication.^19^ Of the original 182 bombing survivors, 103 completed the long-term follow-up interviews; 39 were known to be deceased, 25 could not be located, and 15 declined participation. The follow-up interviews were completed a median of 23 years (range: 21.5-24.3 years) after the bombing. Institutional Review Board (IRB) approval for the longitudinal follow-up study was approved by the University of Texas Southwestern Medical Center (Dallas, Texas USA) IRB, study #092015-024: A 20-Year Follow-Up Study of the Oklahoma City Bombing; initial approval 4/27/16. All participants provided written informed consent before starting the interviews.

Survivors completed open-ended, non-directed interviews asking them to describe in their own words their personal experience of the bombing and its effects on their lives. They were allowed to provide as much or as little discussion as they wished. The interviews were audio recorded and professionally transcribed.

### Data Analysis

Characterization of participant characteristics and testing of multivariate models to identify characteristics associated with study attrition were conducted using SAS 9.4 (SAS Institute; Cary, North Carolina USA).

ATLAS.ti qualitative software (ATLAS.ti Scientific Software Development, GmbH; Berlin, Germany) was used for this study’s data analysis. For the analysis, one researcher read the text and identified 12 main themes in it. Sections of the qualitative data next were rated by pairs of researchers, and their ratings were compared to achieve excellent inter-rater reliability for each theme as defined by a Cohen’s kappa statistic of ≥.80. Themes achieved kappa values ranging between .88 and 1.00. As part of inter-rater reliability procedures, detailed inclusion/exclusion criteria were developed and refined for each theme. Any discrepancies in ratings and definitions of themes were resolved through discussion between raters and final consensus reached through decision by the principal investigator. After attainment of excellent inter-rater reliability, individual raters coded all of the qualitative text into the themes, coding multiple themes as appropriate for individual text passages, yielding a total of 4,958 total coded items.

The 12 themes included: (1) Location (2%; n = 107 items); (2) Bombing Experience (17%; n = 825 items); (3) Initial Actions (11%; n = 522); (4) Injuries and Medical Care (18%; n = 911); (5) Assistance Received and Given (9%; n = 468); (6) Losses (2%; n = 112); (7) Workplace Issues (6%; n = 302); (8) Authorities and Media (4%; n = 192); (9) Relationships (3%; n = 154); (10) Coping (8%; n = 417); (11) Making Meaning/Perspectives (7%; n = 348); and (12) Thoughts and Feelings (12%; n = 600). The volume of the qualitative material necessitated grouping and subdivision of the themes into subsections. Because of the large volume of the qualitative material collected, this article will provide details of only three themes: Injuries and Medical Care; Assistance Received and Given; and Losses.

## Results

At least 39 of the 182 baseline participants were found to be deceased at follow-up. Of those not known to be deceased, 103 (72%) completed the longitudinal follow-up study. The follow-up sample included 57 (55%) women, 91 (88%) non-minority racial/ethnic membership, with a mean (SD) age of 39.0 (SD = 9.1) years, 35 (34%) with college degrees, 100 (97%) with current employment, 71 (69%) who were married, 87 (84%) with bombing injuries, and 48 (47%) who contemplated their death in the disaster. A multiple logistic regression model predicting completion of follow-up interviews (dependent variable) was tested using independent covariates entered simultaneously into the model including baseline variables of sex, age, minority race/ethnicity, college education, and married status, and known deceased status at follow-up; only older age was significantly associated (df = 1, β = .05, SE = .02, Wald χ^2^ = 5.57, P =.018) with noncompletion of follow-up interviews.

One noteworthy observation was that many of the survivors cried through part or all of the interviews, demonstrating considerable emotion, yet they described the interview experience as generally cathartic, positive, and important for the historical material the survivors could provide. Table 1, Table 2, and Table 3 display the numbers of coded items and subdivisions of their content in each of the three themes, respectively. The content of the qualitative material in the themes is briefly summarized in text and relevant illustrative quotes are provided in Table 4, Table 5, and Table 6.

**Table 1.** Injuries and Medical Care Theme and Groupings of Items Within Them

	Column Totals (n of items coded)				
Physical Injuries				450	
Joints			19		
Lower Extremities			40		
Arm and Hand Injuries			30		
Spine, Neck, Back			25		
Lacerations/Bleeding/Stitches			110		
Embedded Glass/Shrapnel			37		
Burn/Electrical Injury			7		
Head and Facial Injuries			128		
Bruised			8		
Cardiopulmonary			8		
Not Specific			38		
Neuropsychiatric Effects				219	
Mental/Psychiatric/Psychological/Emotional			211		
*Unreal*		27			
*Psychiatric (General)*		13			
*Psychological/Emotional Resiliency*		1			
*Posttraumatic Stress*		123			
*General Posttraumatic*	12				
*Flashbacks/Intrusive Memories*	19				
*Avoidance/Numbing*	33				
*Hyperarousal*	59				
*Depression/Anxiety*		34			
*Suicidality*		3			
*Sleep*		8			
*Substance Use Problems*		2			
Cognitive Problems			8		
Uninjured				19	
Medical Care				139	
Transport to Hospital			35		
Emergency Room Assessment and Care			45		
Hospitalized			35		
Surgery			24		
***Total of Above***					***827***

**Table 2.** Assistance Received and Given Theme and Groupings of Items Within Them

	Column Totals (n of items coded)			
Assistance Given/Received at Bombing Site			282	
Inside Bombing Site		121		
*Help Received*	47			
*Help Given*	74			
Going Back In/Came in to Help	13			
After Getting Outside		142		
*Help Received*	80			
*Help Given*	63			
Volunteer Assistance	5			
Formal Medical Assistance Received			72	
Assistance Received/Given by Community			46	
Help Received by Community		34		
Help Given by Community		12		
Assistance Received/Given by Family/Friends			35	
Assistance Received		34		
Assistance Given		1		
Assistance Received/Given by Workplace			32	
***Total of Above***				***467***

**Table 3.** Losses Theme and Groupings of Items Within Them

	Column Totals (n of items coded)		
Loss of Life		84	
Economic/Financial Losses		12	
Property Losses		100	
Personal Belongings	19		
Clothing/Glasses	13		
Automobile	30		
Living Space	9		
Work Space	21		
Other and General Losses	8		
***Total of Above***			***196***

**Table 4.** Illustrative Quotations Regarding Injuries and Medical Care

Physical Injuries
My scalp was not attached to my head, and it scared me.
[Thrown] face first into the…wall of the building, and that impact fractured my skull.
We picked her up, and…the left side of her face was gone.
He didn’t move his hand off his face was because he was afraid his nose would fall off.
It looked like I was looking through red sunglasses because of all the blood…in my eyes.
I’ve got a piece of glass in my right eye, so I’m blind in my right eye.
My arm was…lacerated…wide open; I could see…to the bone across my biceps.
That glass was just like pellets out of a shotgun…blasted me on the right side of my body.
The glass [from my office windows blowing in] nailed my dress to me.
I was scared to death I was going to bleed to death.
Concrete was up to my waist, so I had to remove [it from] my legs…. My legs were bloodied…. I looked at my left leg, and I could see my bone hanging out of my knee.
I had head and face injuries from the fluorescent light fixture that came down from the ceiling, cut my ear in half…then the arm and the hand injuries were from the concrete wall falling over, and then my back was full of shrapnel.
Part of the Ryder truck cut both my legs to the bone.
A piece of shrapnel severed my Achilles tendon in my right leg.
I was electrocuted… some wires had fell down on me, because I could actually remember hearing the wires arcing and sparking and cracking in the background… those two little burn marks was where the entry and exit was of the electricity going through me.
**Mental/Psychiatric/Psychological/Emotional Effects**
[Hyperarousal responses]: lightning…and anything loud above my head.
During the two weeks after the bombing, feeling depressed [and] jumpy.
**Medical Care**
My blood pressure was 50 over 0, and basically, I was bleeding out, and that if I hadn’t have gotten to the hospital when I did, I probably would have died from blood loss in another five minutes. So quick and effective first responder support was absolutely the only reason that I survived.
I had probably a couple hundred glass shards throughout my body…like I’d been shot with a shotgun blast…. They were picking each one of those out [with] tweezers.
They took me directly into a surgical suite…about five doctors worked on me for 5.5 hours …. My left lung was punctured. They got me breathing.…. I was kind of semiconscious, but I was afraid that I was going to quit breathing again and die…[the] surgeons were pulling stuff out of me…filled up a trash can with shrapnel, and periodically an artery…would pop open and spray blood, and they would clamp it off, and then…give me more blood.
A priest came in… if I was going to die, I didn’t want to give him my name… the nurses said, “We’re not going to let you die.” I said, “What if I stop breathing again?” And they said, “Well, then we’re going to put you on life support and breathe for you.”
I remember waking up thinking I was like in the morgue, in a casket, because of the floral smell and everything, but yet I couldn’t really just jump up and assess everything. I was still pretty doped up. So I remember thinking, you know, I was dead or something.

**Table 5.** Illustrative Quotations Regarding Assistance Received and Given

Assistance Given/Received at Bombing Site
I heard one of my coworkers say, “Okay, we’re going to…come in and get you.”
He put me on the desk and started to administer first aid, and I remember him telling me that I looked pretty bad and that he was going to help me.
He says, “I want you to get up,” and I said I’d try. He lifted me up, and he got me going.
The guys found a chair, and they put me in a chair, and they were able to carry me out.
We were putting people…on the ground, in the street outside the building…trying to get people taken care of.
He and I decided that he would stay with those three ladies and make sure they got out, and I was going to go back and look for people.
She wanted to go get her baby, and I said, “You cannot walk on all this glass without shoes.” So I went and got her shoes.
After we got several kids out of there, I went back over to the Murrah Building and started digging through rubble again.
We started…pulling all the Kleenexes we had in our purses for people who were sitting out there and who obviously had glass in their face and were sharing our Kleenexes.
They went room to room…and grabbed all the women’s purses out [to take to their owners]. They weren’t supposed to be in there, but they sneaked in.
**Formal Medical Assistance Received at Site**
I remember getting my head bandaged.
There was a medical person that came and gave me some gauze, and by that time I had pretty much stopped bleeding.
They had go-karts that was around there to pick up people to take them there so they wouldn’t have to walk if they were injured.
The triage people were trying to run with my stretcher.
He got me to the first ambulance that pulled up. The ambulance was full…they couldn’t lay me down anyway, but the people on the ambulance helped me up.
**Assistance Received/Given by Community, Family, Friends, and Workplace**
Incredible outpouring of the community to help people. My family was helped. We got assistance from different organizations and just friends and neighbors.
People that you would not expect…came up and hugged us and gave us their blessings…. I just couldn’t get over the feeling of love and concern that was in the air.
I stood there in line from like 2:00 in the afternoon until almost 8:00 just to give blood, because I felt like that’s the only thing I could do to help.
We went down and helped take food to the workers.
I spent a lot of time going around to people’s homes and making sure they were okay, especially the people who got laid off.
With my concussion, they wanted her to wake me up and check my eyes and all that, and she stayed with me that night.
My wife would take me to the doctor and bring me home and treat my wounds and all those kinds of things so I would not have to be hospitalized.
One of the investigators of our department went from hospital to hospital and tracked us down and then called our family.

**Table 6.** Illustrative Quotations Regarding Losses

Loss of Human Life
There was only four of us that were there at the time that survived. There were…six or more, maybe, in our office that perished…. The others probably were not coming out, because we saw the front of the building and… [realized that] they were underneath all of the debris.
We lost…eight people that I closely worked with.
I just started thinking about, you know, those people that I used to see, and I’m sure, you know, they’re all gone.
I lost some very dear friends in that bombing.
I’ll never, ever forget the people who died that day, and I keep up with some of their families.
You almost go back to the last time you saw them in the building and have to remember that they’re no longer going to come to work.
**Economic/Financial Losses**
I was making a whole lot of money running the whole place and couldn’t find a job that would pay me anything.
I go from having a job, being a supervisor on day, to having no job.
We took a big cut in our salary.
You know how hard it is to pay your car payments and your house payment when you don’t have a job because you lose it.
Control had been taken from my life.
**Property Losses**
None of the women had any of their purses. They didn’t have the keys to their house. They didn’t have any ID.
We had no car keys…our purses were in there.
My computer and everything in the whole room was completely destroyed, all my law degree certificates.
We were able to get some of the personal belongings out of the building…. The favorite thing that I had was a picture of my son’s handprint, and that particular picture that he had made when he was little, it had gotten shattered and damaged in the bombing.
[My car] was totaled. The tires were blown out and…the windows were blown out, and the roof was pressed down into the passenger compartment, the I-beams in there twisted like pretzels.

Injuries and Medical Care was the largest theme in terms of numbers of coded items, containing >900 items (18% of the total qualitative content of the 12 themes). Consistent with the 84% of survivors injured, this theme included large numbers of coded items pertaining to physical injuries (Table 4). Many injuries were sustained to head and face; upper and lower extremities with crush injuries and fractures; spine, neck, and back injuries; embedded glass and shrapnel; bruises; cardiopulmonary injuries; and electrical injuries. Table 1 shows enumeration of the number of coded passages across the types of injuries sustained.

Most of the injuries described were to the head and face, especially lacerations. Several survivors described glass embedded in eyes, blindness, or loss of one or both eyes. Table 4 includes quotations providing detailed descriptions of severe injuries to head, nose, face, and skull with skull fractures and major facial/head appendices severed or missing. Extensive lacerations from head to toe, causing considerable blood loss, were attributed to high-velocity flying glass from large windows and glass building exteriors. Shrapnel injuries had many mentions. A number of comments described severe injuries to upper and/or lower extremities. A number of survivors had to have their lower extremities amputated to free them from heavy rubble crushing and trapping their limbs. Some electrical and burn injuries were described.

Neuropsychiatric effects of the bombing constituted only approximately one-half of the amount of coded content of personal injuries, most of which was psychological rather than neurocognitive, with the largest portion consisting of posttraumatic stress responses. The major portion of the posttraumatic symptom content was hyperarousal. Environmental stimuli after the bombing experience precipitated hyperarousal responses. These fears corresponded to avoidance responses that included several mentions of avoidance, such as being unable to return to the site of the bombing, go to the memorial museum, or be around people. There was only one mention of feelings of numbness. There was considerable discussion of unpleasant reminders of the bombing, including having to return to the workplace, several mentions of nightmares of the bombing or reminders of it, and general sensations of reliving the bombing again. Only four comments indicated personal development of posttraumatic stress disorder (PTSD) and three additional comments mentioned PTSD in others; therefore, most of the material in the posttraumatic stress section of the qualitative data appeared to relate to posttraumatic distress of nonpathological proportions rather than to formal PTSD symptoms and disorders. Illustrative quotations related to these neuropsychiatric consequences can also be seen in Table 4.

Comments reflecting depression and anxiety were far less represented than comments reflecting posttraumatic stress in numbers of coded items (Table 1). The descriptions of depression and anxiety ranged from transient to severe and long-lasting, such as extensive weight loss within the first month and several years of inability to concentrate and self-imposed confinement to one’s residence. There were very few mentions of suicidality or substance abuse problems.

Fewer comments were made on medical care received than on physical injuries, and illustrative quotations are provided in Table 4. Transportation to medical care required nontraditional approaches to saving lives, such as taking several survivors in a single ambulance. At least one survivor was impressed with how quick the transportation to the hospital was, how efficient the hospital triage was, and how bumpy the ride was on the hospital gurneys. Survivors said they received hundreds of sutures on several body areas, so many that the physicians lost count and their hands were sore, and several physicians worked on their different injuries simultaneously. One comment described use of a staple gun to reattach the scalp to the head. Glass was meticulously pulled from skin and from eyes. Survivors described being in a great deal of pain, feeling like their hospital experience was a dream, and having confused episodes of thinking there was someone hiding under their hospital bed. Several surgical procedures were mentioned, with a few comments describing dramatic confrontations with potential for death. One survivor described being discharged from the emergency room after being sutured, but “my spinal and brain fluid had leaked out onto my pillow, so I’d had a much more severe injury than they thought, and I had to go for emergency brain surgery for a shattered skull and subdural hematoma.”

Table 5 provides illustrative quotations included in the theme of Assistance Given and Received. Survivors provided assistance to others inside the bombed buildings, helping to rescue others by removing them from the rubble and carrying them out. One survivor described a man who “was in shock. I had to slap him, to shake him.” After escaping the bombing zone, a number of survivors went back in to help others: “Some people went back in…to see if…everyone was out of the building.”

Once outside, a number of survivors were cold and shaking, and several people provided coats and blankets to them. Others provided transportation to take people home, took care of children until their parents could arrive, and made phone calls for survivors to inform their loved ones that they were alive. Formal assistance was provided by medical personnel at the site.

People described an outpouring of community assistance and volunteerism. One survivor summed up this phenomenon as: “Our city was just incredible after the bombing. You hear all kinds of stories about the things people did for others. Family and friends provided assistance, and some employers were very helpful.” Another expressed this community assistance as: “Just seeing people come together to help other people, and the outpouring and the support, was really just incredible, just unbelievable.”

There were positive comments about the value of the research: “If we keep talking to the professionals and keep participating in the studies, that may be part of our legacy…[if] we help the next man or woman or child who went through something horrible, and that is what I call one of my highest duties;” “The academic and medical and scholarly studies…are going to help the people tomorrow, and I think the work you folks are doing is important…. Thank you…. I am living proof that when you call us in and talk to us and things you say to us do help.”

Many types of losses were discussed in the theme of Losses (Table 6 shows illustrative quotations). Losses included loss of life, economic/financial/career losses, and property loss (personal belongings, automobiles, living space, and work space).

The section on loss of life included comments on the deaths of more than one-half of the coworkers in their office, including 35 lives in just one office and another office with 18 of 33 coworkers killed. The comments on the loss of lives were painfully described, with descriptions of how much the survivors to this day still miss their many coworkers lost in the bombing.

The majority of comments were about economic losses and losses of property. There were nine comments by women who lost their purses in the building and several comments about loss of clothing, glasses, and other personal effects.

## Discussion

This is one of the longest prospective longitudinal studies ever conducted to obtain qualitative descriptions of personal injuries, medical care, assistance given and received, and losses among highly trauma-exposed survivors of a terrorist bombing. The main injuries described were to the head and face including loss of eyes or eyesight, lacerations from flying glass, and also shrapnel, fractures and amputations, and electrical injuries.

Emotional effects consisted predominately of posttraumatic stress reactions (intrusive recollections, hyperarousal, and avoidance/numbing) consistent with results of quantitative studies usually finding posttraumatic stress symptoms and PTSD to be the most prevalent disaster-related psychiatric sequelae.^19–22^ Feelings of depression and anxiety were also described, but few survivors reported diagnoses of PTSD or major depressive disorder. There were many descriptions of unreal feelings, but not of substance abuse problems. Numerous coded passages described extensive and dramatic medical care. Many coded passages described survivors inside the bombed buildings assisting others, risking their own lives and some returning inside to help. More comments regarding experiences outside of the building described assistance received than provided. Numerous comments were made about community and other outside assistance, and descriptions of this assistance were poignant. Some comments were positive about the research. Relatively few of the 4,958 passages coded in this study discussed loss of human life despite the 168 deaths in this disaster, but their descriptions were intense. Many comments indicated property (especially purses and automobiles) and financial losses incurred in the bombing.

In many regards, the qualitative material speaks for itself in that even nearly a quarter century after the bombing, their memories were still vivid, graphic, and evocative. Anecdotally, many of the survivors cried through part or all of the interviews, yet finding the interviews to be a positive experience. Several thanked the researchers for conducting this work, expressing hope that it would help others in the future by getting their stories out and expressing gratitude for the research.

This study’s qualitative findings are relatively consistent with the results of a 14-year post-9/11 study of members of a registry of affected individuals, 44% of whom were injured, especially with fractures and dislocations, burns, and head injuries.^1^ Long-term MH findings of the existing quantitative research on disaster survivors^2–17^ resonate with this long-term study’s qualitative findings. The 9/11 study^1^ found that emotional distress and employment difficulties arising from the disaster generally improved across time; for some, however, these problems continued for long periods, changing people’s lives forever. The findings of the current study appear to be generally consistent with the 9/11 findings common to both studies. Attempts to compare the long-term findings with results in the voluminous acute post-disaster literature are fraught with confounding of very different perspectives, time frames, and research methods. Unfortunately, little research has been conducted from the vantage point of decades after disaster, and almost no qualitative data from this time frame for comparison with the current study’s findings.

A central strength of the current study is its prospective longitudinal, post-disaster follow-up, allowing survivors to describe their experiences and outcomes over a very long period of time spanning the time of the bombing to almost a quarter of a century later. The number of longitudinal disaster studies of this duration is limited; few focused on terrorist events, and even fewer provided qualitative data, indicating the importance of the current study’s data. Another strength is the random sampling with 71% baseline participation rates and 72% follow-up of non-deceased survivors, with only greater age associated with follow-up attrition. The research design involving open-ended qualitative interviews is another strength, with participants recounting their disaster experience in their own words without specific direction limiting potential topic areas. This study’s open-ended interviews were audiotaped and professionally transcribed rather than the simple paraphrasing of responses to questions recorded by hand by interviewers used in earlier research on survivors of this disaster that may have missed important information and introduced error.^18,23^ Using these qualitative methods, this study provided extensive detail of survivor disaster experiences pertaining to personal medical and emotional effects, medical care, assistance, and losses from the survivors’ own point of view nearly a quarter century after the bombing.

The results of this long-term qualitative study demonstrated that survivors recall their injuries, medical care, assistance received and given, and losses with vivid clarity and strong emotions, even nearly a quarter century later. This is of great importance to disaster emergency response, given how clearly and emotionally the survivors appear to recall the experience, carrying the memories with them for decades. This finding speaks to the critical importance of effective management of the disaster response and early care for the survivors, as the effects may be long-lasting and shape the rest of their lives.

Despite strong effects on people’s lives nearly a quarter century later and many responses indicating continuing strong emotions related to the bombing, the qualitative content suggested that lasting formal psychopathology was not a central concern. Given that 34% of this sample were found to have PTSD and 45% any disorder by diagnostic interviews six months after the bombing,^19^ this finding could reflect either recovery from psychiatric illness or lack of spontaneous discussion of psychiatric illness.

The discussion of loss of the lives and financial and property losses decades after the bombing introduces the possibility that psychosocial interventions might prove helpful even this long after the disaster. Survivors with active struggles with these losses could be identified for provision of psychosocial interventions, including counseling and bereavement therapy, as appropriate.

A direction for future research is to compare this long-term study’s findings to qualitative data collected in the early post-disaster period, and to compare qualitative findings across different types of disasters and populations, both of which were beyond the scope of this article. Future research might also compare qualitative findings with quantitative personal characteristics, especially PTSD and other disaster-related psychopathology. Finally, application and testing of interventions among individuals expressing distress decades later is needed to determine the benefit of interventions in this late time frame.

## Limitations

The long-term duration of the data collection since the disaster may also represent a potential limitation to the study, allowing for corruption of memory. Because only one disaster cohort of one disaster type (terrorism) in one site was included in this study, the findings may not generalize to other disasters, such as the 1986 Chornobyl disaster resulting in nuclear radiation exposure;^24^ the 1982 Times Beach, Missouri chemical exposure to dioxin;^25^ and exposure to toxic dust among 9/11 rescue workers and survivors leading to long-term cancer risk and severe pulmonary disease.^26,27^ Thus, additional very long-term follow-up research such as this study is needed to verify and expand these findings with other disaster populations. Further research is needed to identify personal characteristics of survivors that predict such long-term findings.

## Conclusion

This is one of the longest prospective longitudinal, qualitative studies ever conducted with highly trauma-exposed survivors of a terrorist bombing, nearly a quarter century after the disaster. It generated long-term systematic information from open-ended descriptions of disaster survivors regarding their medical and psychosocial outcomes, assistance received and given, and disaster-associated losses. Survivors recalled their injuries, medical care, assistance received and given, and losses with vivid clarity and strong emotions, even nearly a quarter century later. These findings confirm the critical importance of effective early management of the disaster response and care for the survivors, because the disaster had long-lasting effects, shaping the rest of their lives. Terrorist incidents continue to occur globally with considerable frequency, generating increasing populations of trauma-exposed survivors with many long-term needs.
